# Knowledge, attitudes, and practices regarding chikungunya fever among healthcare workers: a cross-sectional study in Sichuan Province, China

**DOI:** 10.3389/fpubh.2025.1729173

**Published:** 2026-01-13

**Authors:** Jun Luo, Xueshuang Liu, Kang Chen, Wenshuang Wei

**Affiliations:** 1 Ministry of Basic Medical Education, Dazhou Vocational College of Chinese Medicine, Dazhou, China; 2 Department of Emergency, The Second People’s Hospital of Linshui, Guangan, China

**Keywords:** knowledge, attitudes, practices, chikungunya fever, healthcare workers, China

## Abstract

**Objective:**

To assess the knowledge, attitudes, and practices (KAP) of healthcare workers (HCWs) regarding Chikungunya fever and its influencing factors in non-endemic Sichuan Province, China.

**Methods:**

A cross-sectional online survey was conducted among 312 HCWs in August 2025. Data were collected using a structured KAP questionnaire and analyzed with descriptive statistics, chi-square tests, binary logistic regression, and correlation analyses.

**Results:**

The great scores rates for knowledge, attitudes, and practices were 60.90%, 65.38%, and 40.06%, respectively. Knowledge was positively correlated with attitudes (*r* = 0.403, *p* < 0.001), and attitudes with practices (*r* = 0.661, *p* < 0.001). Knowledge was significantly higher among physicians (OR = 1.607) and secondary hospital staff (OR = 1.901). Senior professional title and 5–10 years of work experience were associated with more positive attitudes. Although most HCWs recognized the importance of prevention (94.88%) and had high learning willingness (90.06%), practical performance was low—only 34.97% regularly conducted public health education.

**Conclusion:**

Significant gaps exist in core knowledge and its translation into practice among HCWs in non-endemic areas. Targeted training, especially for nurses and primary care providers, is urgently needed to enhance outbreak preparedness.

## 1 Introduction

Chikungunya fever is caused by the Chikungunya virus (CHIKV), which is transmitted by Aedes mosquitoes (1). It is characterized clinically by acute fever, severe joint pain, and rash. In addition, a high percentage of patients may progress to a chronic infection in joints characterized by intense pain and deformities, persisting from several months to several years, which poses a burden on patients’ quality of life and the healthcare system (2, 3).

While the virus was first identified in Tanzania in 1952 (4), its global range has expanded dramatically due to globalization, climate change, and vector spread, making it a major emerging public health threat in over 60 countries across Africa, Asia, the Americas, and Europe (2, 3, 5). Since 2008, when imported cases of CHIKV were first detected in China (6), there have been many local outbreaks caused by imported cases in border and coastal provinces such as Yunnan and Guangdong from 2010 (7, 8).

In 2025, an outbreak of chikungunya fever occurred in Guangdong Province, China, and as of July 26, 4,824 local cases had been reported in the province for the current year (9). To contain further spread, health authorities activated the rapid coordination framework originally established during the COVID-19 pandemic for chikungunya control. Primary healthcare providers adhered to the “early reporting, early isolation, and early management” principles outlined in China’s National Infectious Disease Response Framework, facilitating timely case identification. Notably, a primary care practitioner recognized a cluster of similar symptoms and promptly reported it, reducing the interval between the first suspected case and CDC notification from 30 days during the 2010 Dongguan outbreak to just 11 days in 2025, a substantial improvement in response efficiency (10).

Sichuan Province, a populous transportation hub in southwestern China with a hot, humid climate suitable for the CHIKV vector *Aedes albopictus*, remains at high risk for local outbreaks following case importation. The recent dynamics in Guangdong underscore the critical need for heightened preparedness in at-risk yet currently non-endemic areas like Sichuan.

The Knowledge-Attitude-Practice (KAP) model serves as a fundamental framework for analyzing and shaping health-related behaviors (11). Initially applied in family planning, it is now extensively used in public health to reveal population perceptions and behaviors regarding disease transmission and control, which in turn influence outbreak patterns and distribution (11, 12). Medical personnel, as the first line of defense for CHIKV monitoring, diagnosis, reporting and management, the level of KAP of CHIKV directly determine the efficiency of early identification of imported cases and the effect of emergency response to an outbreak (13).

Given the lack of a vaccine and targeted therapeutics, management of CHIKV relies on public health education and vector control. While KAP studies among HCWs exist for other vector-borne diseases like dengue and malaria (14–17), there is a notable lack of such studies on CHIKV targeting HCWs in mainland China. Compared to their counterparts in endemic areas, HCWs in non-endemic regions like Sichuan have less clinical exposure to CHIKV and may lack knowledge and experience in its diagnosis and treatment, potentially increasing the risk of management failures during an outbreak (18, 19). Based on this, this study aimed to assess the knowledge, attitudes, and practices of healthcare workers in Sichuan Province, and to provide a basis for further planning of education for HCWs on CHIKV and its prevention and control.

## 2 Materials and methods

### 2.1 Study area

Located in southwestern China, Sichuan Province lies between 97°21′-108°33′E and 26°03′-34°19′N. Sichuan Province covers an area of approximately 486,000 km (2) and has a population of over 83 million. The province includes 21 prefecture-level divisions, ranging from densely populated urban centers (e.g., Chengdu) to rural and mountainous areas. Sichuan Province is characterized by diverse climatic conditions due to its varied topography. The annual average temperature ranges from 16 °C to 20 °C, while the average annual precipitation varies between 900 mm to 1,200 mm across different regions. The western mountainous areas experience cooler temperatures and higher rainfall, while the Sichuan Basin has a more humid subtropical climate with warmer conditions. In general, the warm temperatures and high humidity of Sichuan Province provide a suitable environment for Aedes mosquitoes, the primary vector of chikungunya fever.

### 2.2 Study design

This cross-sectional study was carried out from August 1 to August 30, 2025, involving healthcare professionals in different health facilities across Sichuan Province. A convenience sampling method was employed for participant recruitment. The survey was conducted anonymously via WeChat (version 8.0.61) to encourage candid responses, a platform which inherently restricts each unique user account to a single submission to prevent duplicates and ensure data integrity. The inclusion criteria for this study were: (i) licensed healthcare workers (physicians/nurses); (ii) currently employed in healthcare facilities of Sichuan Province; (iii) required to have at least 6 months of clinical experience; (iv) willing to participate voluntarily. Out of 319 questionnaires distributed, 312 were valid (97.81%). Seven responses were excluded due to missing demographic data, resulting in a final sample of 312.

### 2.3 Data collection

Data was collected by using a self-administered online questionnaire, which was constructed following an review of relevant literature and Technical guidelines for the prevention and control of Chikungunya fever (2025 Edition) (14, 16, 20, 21). To ensure content validity, the questionnaire was reviewed by three experts within our research team, including epidemiologists and infectious disease specialists. The questionnaire was composed of four sections: (1) demographic and job-related information; (2) knowledge of chikungunya fever; (3) attitudes toward Chikungunya fever; (4) Chikungunya-related practices.

The demographic and job-related information section, including 8 questions, gathered data regarding age, gender, education, job, workplace, professional title, department and years of experience.

The knowledge assessment section encompassed 12 questions on pathogen, symptoms, transmission, clinical management, prevention and control of the disease. The knowledge section primarily consisted of five-choice questions requiring participants to select the single correct answer, while a subset of items employed a three-option format (yes/no/do not know). Each correct response was scored one point, with incorrect answers receiving zero points, yielding a total possible knowledge score between 0 and 12.

The attitudes toward chikungunya fever were assessed by five single choice and one multiple choice, for example, “Do you think the risk of local Chikungunya fever transmission is high in your city.” Participants rated their responses using a 5-point Likert scale (i.e., 1 = very disagree; 2 = disagree; 3 = not sure; 4 = agree; and 5 = very agree). For multiple choice questions, one point for each option. Total attitudes scores ranged from 6 to 32, with higher values indicating more positive attitudes.

The section on chikungunya-related practices consisted of 4 items, evaluating three key domains: clinical practices (frequency of travel history documentation and prevention education delivery), training engagement (participation in continuing education), and preventive actions. The first three questions are single-choice items evaluated on a five-point scale. The last question is a multiple-choice item, and each of the other options selected scores 1 point, resulting in a total score range of 1–8. Total actions scores ranged from 6–23.

Consistent with previous KAP studies using 70–75% cutoffs (14), this study adopted a 70% benchmark to dichotomize participants into ‘poor’ and ‘good’ KAP categories. Respondents scoring above this threshold were deemed to have adequate KAP levels.

### 2.4 Statistical analysis

Statistical analysis was carried out with the Statistical Package for the Social Sciences (IBM SPSS Statistics for Windows, Version 27). Frequency and percentage were employed to describe the categorical variables, while mean±standard deviation (SD) percentage were used to report continuous variables. The chi-square test was performed to compare the scores of the knowledge, attitudes, and practices (KAP) regarding chikungunya fever between different groups. Logistic regression analysis was conducted to determine the affecting factors. A value of *p* < 0.05 was considered statistically significant.

## 3 Results

### 3.1 Study population characteristics

A total of 312 healthcare professionals successfully filled in and completed the questionnaire, of whom 68.27% were female. The mean age of all the participants was 32.71 ± 8.45. The largest group of participants were those aged 19 to 30 years, accounting for 47.76%. A majority (90.38%) held a junior college or bachelor’s degree. Additionally, 55.77% were physicians and half of the participants (51.28%) worked in primary hospitals. Regarding professional title, 34.62% had no professional title and 29.49% held an intermediate-level title. 38.14% worked in internal medicine departments, and 39.10% had 5–10 years of work experience (Table 1).

**Table 1:** Socio-demographic characteristics of the study participants.

Variable	*N*	%
Age (Years)		
19–30	149	47.76
31–40	117	37.50
41–50	32	10.26
51–60	14	4.49
Gender		
Male	99	31.73
Female	213	68.27
Education		
Junior high school	3	0.96
Senior/vocational high school	16	5.13
Junior college/bachelor’s degree	282	90.38
Master’s degree or above	11	3.53
Occupation		
Physician	174	55.77
Nurse	138	44.23
Workplace		
Primary hospital	160	51.28
Secondary hospital	120	38.46
Tertiary hospital	32	10.26
Professional title		
None	108	34.62
Junior	86	27.56
Intermediate	92	29.49
Senior	26	8.33
Department		
Internal medicine	119	38.14
Surgery	46	14.74
Public health	29	9.29
Others	118	37.82
Working years		
<5	89	28.53
5–10	122	39.10
11–15	42	13.46
>15	59	18.91

### 3.2 Association between knowledge, attitudes, and practices

As shown in Table 2, the scores of knowledge, attitudes, and practices were 8.68 ± 2.36, 23.98 ± 3.67, and 15.05 ± 4.03, respectively. The great scores in 60.90% of knowledge, 65.38% of attitudes, and 40.06% of practices items. The good scores among the correlation between knowledge and attitudes was found to be significant (*p* < 0.001), suggesting that higher knowledge was associated with a more positive attitude. Similarly, the correlation between attitudes and practices was also significant (*p* < 0.001), indicating that a more favorable attitude was linked to better practice.

**Table 2:** Correlations of knowledge, attitudes, and practices.

Variables	Mean±SD	X1	X2	X3
X1 Knowledge	8.68 ± 2.36	1.000	0.403**	0.279**
X2 Attitudes	23.98 ± 3.67		1.000	0.661**
X3 Practices	15.05 ± 4.03			1.000

**p < 0.001.

### 3.3 Knowledge of chikungunya fever

The vast majority of participants (96.47%) were aware that mosquito vector is the main mode of transmission of chikungunya fever, and more than 85% knew that the disease is caused by a virus (86.54%), is generally susceptible to infection (89.74%), and is distributed in the tropics/subtropics (87.18%), and agreed that mosquito prevention is the key to preventing chikungunya fever (87.50%).

However, there was a significant lack of knowledge about diagnosis and treatment: only 56.09% knew that RT-PCR was the main diagnostic method, 47.44% understood that supportive therapies were the main treatment, only 35.90% were aware of the possibility of lasting immunity after infection, and 80.77% mistakenly believed that there was already a vaccine available. In terms of symptom recognition, about 70% were aware of the incubation period of 3–7 days (70.83%) and atypical manifestations such as vomiting (69.55%), and the awareness rate of infectious disease reporting was 59.62% (Table 3).

**Table 3:** Participants’ knowledge about chikungunya fever (*N* = 312).

Knowledge aspect questions(correct answers)	Number (*n*)	Percent (%)
K1 Virus is the pathogen causing Chikungunya fever	270	86.54
K2 Mosquito is the primary vector for Chikungunya fever transmission	301	96.47
K3 All people are universally susceptible to Chikungunya fever.	280	89.74
K4 The most common laboratory method for diagnosing Chikungunya fever is RT-PCR.	175	56.09
K5 The incubation period for Chikungunya fever is typically 3–7 days.	221	70.83
K6 Vomiting is not included in the common symptoms of Chikungunya fever.	217	69.55
K7 Lasting immunity can be obtained after infection with chikungunya.	112	35.90
K8 The primary treatment for Chikungunya fever is supportive care.	148	47.44
K9 There is no currently a licensed vaccine for Chikungunya fever in China.	252	80.77
K10 The most effective way to prevent Chikungunya fever is avoiding mosquito bites.	273	87.50
K11 Chikungunya fever is most prevalent in tropical and subtropical regions.	272	87.18
K12 A suspected case of Chikungunya fever should be reported online after identification within 24 h.	186	59.62

The chi-square test indicated significant differences in knowledge scores across groups based on occupation and workplace (*p* < 0.05) (Supplementary Table S1). Binary logistic regression further showed that physicians had significantly higher knowledge scores than nurses (OR = 1.607, 95% CI: 1.009–2.560), and staff in secondary hospitals scored higher than those in primary hospitals (OR = 1.901, 95% CI: 1.156–3.127) (Supplementary Table S4).

### 3.4 Attitudes toward chikungunya fever

**Figure 1:**
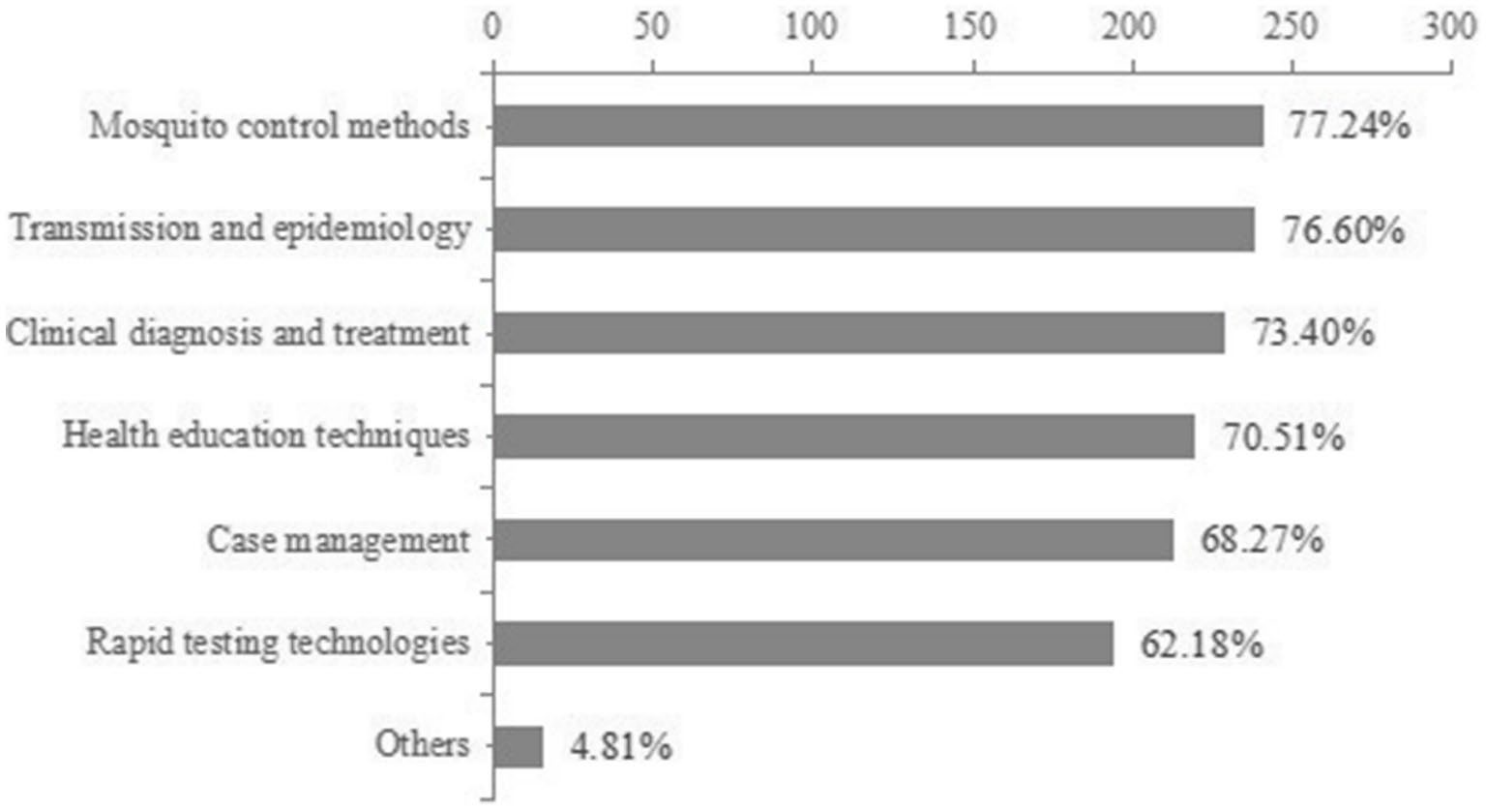
Healthcare workers’ self-reported training interests and knowledge needs regarding chikungunya fever prevention and treatment (*N* = 312). Data are presented as the percentage of respondents expressing interest in each topic area.

67.63% of respondents perceived a low risk of local Chikungunya fever. The vast majority recognized the effectiveness of mosquito control measures (75.64% agreed or strongly agreed) and showed a positive willingness to learn (90.06% were willing to learn on their own initiative). Almost all respondents (94.88%) agreed that it was important for healthcare workers to have knowledge of prevention and control. However, familiarity with the Technical guidelines for the prevention and control of Chikungunya fever (2025 Edition) was generally low, with only 39.43% indicating that they were familiar or very familiar with it. In terms of topics of interest, mosquito control methods (77.24%) and transmission and epidemiology (76.60%) received the most attention, followed by clinical diagnosis and treatment (73.40%) and health education techniques (70.51%) (Table 4; Figure 1).

**Table 4:** Attitudes about chikungunya fever (*N* = 312).

Questions	Answers	Number (*n*)	Percent (%)
A1 Do you think the risk of local Chikungunya fever transmission is high in your city?	Very disagree	63	20.19
	Disagree	148	47.44
	Not sure	79	25.32
	Agree	18	5.77
	Very agree	4	1.28
A2 Do you think mosquito control measures are effective in preventing Chikungunya fever transmission?	Very disagree	6	1.92
	Disagree	9	2.88
	Not sure	61	19.55
	Agree	165	52.88
	Very agree	71	22.76
A3 You are willing to proactively learn about Chikungunya fever prevention.	Very disagree	3	0.96
	Disagree	4	1.28
	Not sure	24	7.69
	Agree	156	50.00
	Very agree	125	40.06
A4 It is important for healthcare workers to master Chikungunya fever prevention knowledge.	Very disagree	3	0.96
	Disagree	3	0.96
	Not sure	10	3.21
	Agree	148	47.44
	Very agree	148	47.44
A5 You are familiar with China’s Chikungunya fever prevention and control technical guidelines (2025 Edition).	Very disagree	6	1.92
	Disagree	54	17.31
	Not sure	129	41.35
	Agree	93	29.81
	Very agree	30	9.62
A6 Which topics about Chikungunya fever prevention interest you? (Multiple choices)	Mosquito control methods	241	77.24
	Transmission and epidemiology	239	76.60
	Clinical diagnosis and treatment	229	73.40
	Health education techniques	220	70.51
	Case management	213	68.27
	Rapid testing technologies	194	62.18
	Others	15	4.81

The chi-square test results indicated that there were statistically significant differences in attitudes among groups stratified by professional title and working years (*p* < 0.05) (Supplementary Table S2). Binary logistic regression analysis further revealed that the attitude scores were higher among senior than those with no professional title (OR = 2.099, 95% CI: 1.016–4.337), and higher among those with 5–10 years of work experience compared to those with less than 5 years (OR = 4.438, 95% CI: 1.627–12.100) (Supplementary Table S4).

### 3.5 Practices related to chikungunya fever

**Figure 2:**
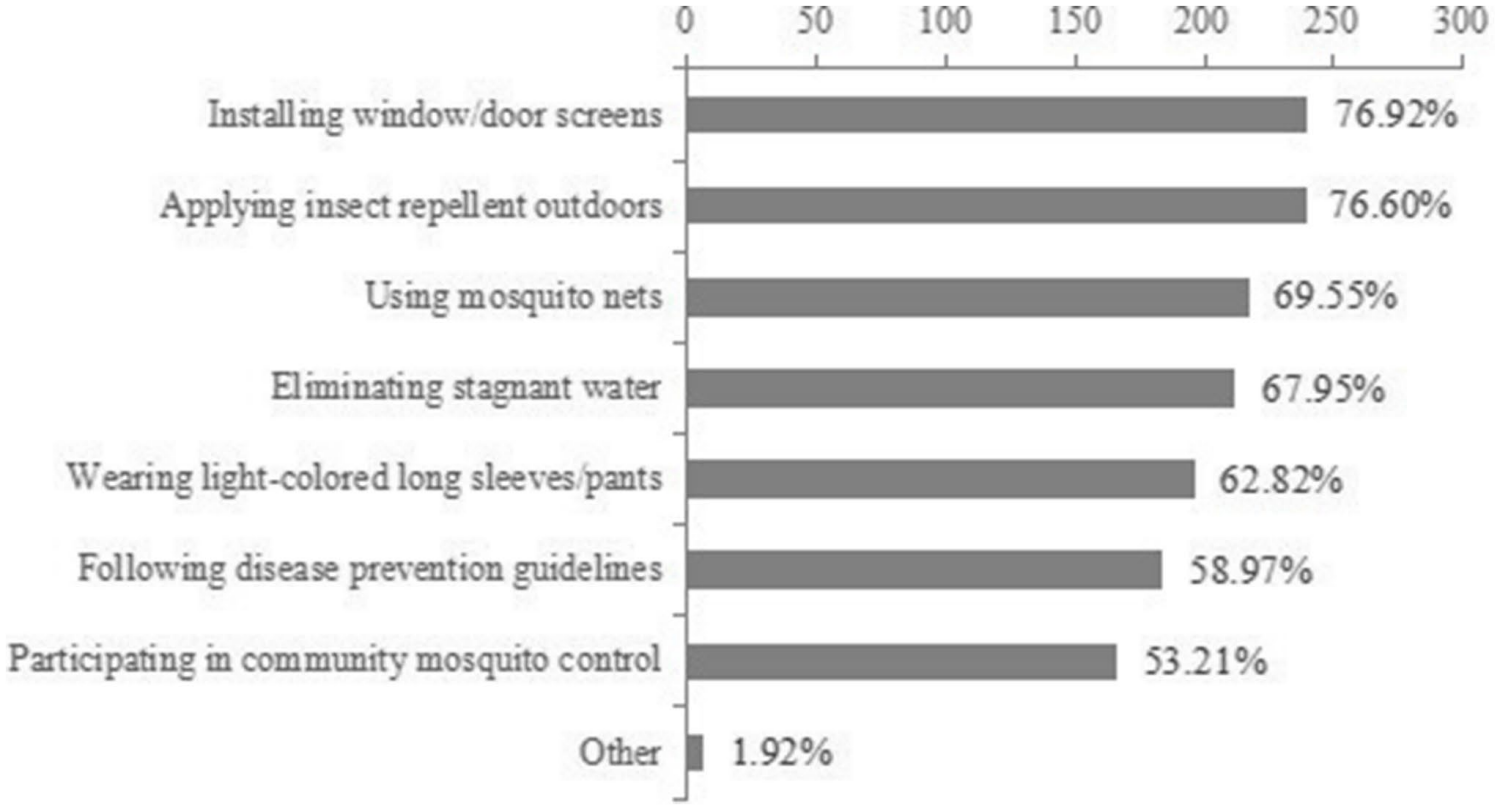
Self-reported preventive measures adopted by healthcare workers against chikungunya virus infection in Sichuan Province, China (*N* = 312). Values represent the percentage of respondents routinely practicing each measure.

Majority (68.59%) were able to ask for travel history frequently or always while attending to patients with fever with arthralgia. Regarding training participation, half (50.96%) had participated in 1–2 training in the past year, 11.54% had participated in 3 or more training, but 28.85% still did not have access to training despite their willingness to do so. The frequency of health education activities was relatively decentralized, with only 34.97% being able to conduct public education frequently or consistently. Preventive measures with high adoption rates included installing window screens (76.92%), using mosquito repellents (76.60%) and removing stagnant water (67.95%), while the implementation rates of unified community mosquito control (53.21%) and guideline compliance (58.97%) were relatively low (Table 5; Figure 2).

**Table 5:** Practices about chikungunya fever (*N* = 312).

Questions	Answers	Number(n)	Percent(%)
P1 When encountering a patient with fever and joint pain, how often do you ask about recent travel history (e.g., visits to endemic areas)?	Never	9	2.88
	Rarely	35	11.22
	Occasionally	54	17.31
	Frequently	123	39.42
	Always	91	29.17
P2 In the past year, how many times have you attended Chikungunya fever prevention training (e.g., lectures, online courses)?	Do not know	18	5.77
	0 times (unwilling to attend)	9	2.88
	0 times (willing to attend)	90	28.85
	1–2 times	159	50.96
	≥3 times	36	11.54
P3 Have you conducted Chikungunya fever prevention education for patients or residents?	Never	30	9.62
	Rarely	80	25.64
	Occasionally	96	30.77
	Frequently	81	25.96
	Always	25	8.01
P4 Which preventive measures have you recently adopted? (Multiple choices)	Installing window/door screens	240	76.92
	Applying insect repellent outdoors	239	76.60
	Using mosquito nets	217	69.55
	Eliminating stagnant water	212	67.95
	Wearing light-colored long sleeves/pants	196	62.82
	Following disease prevention guidelines	184	58.97
	Participating in community mosquito control	166	53.21
	Other	6	1.92

The results of the chi-square test showed that none of the differences in the practice scores of people with different socio-demographic characteristics were statistically significant (Supplementary Table S3).

## 4 Discussion

In this study, we conducted the first cross-sectional survey on the knowledge, attitude and practice (KAP) level of medical personnel in Sichuan Province, China, a non-Chikungunya endemic area. The results of the study showed that despite the overall positive attitude of local health workers toward chikungunya fever prevention and control, there was a significant shortfall in core knowledge and an inefficient translation of attitudes into practice. This disconnect between the KAP dimensions has practical public health implications, especially in the context of the continued increase in imported chikungunya fever cases and the ecological environment of Sichuan Province, which is prone to local transmission.

This study found that the good knowledge regarding chikungunya was 60.90%, higher than other studies (22, 23). Slightly higher knowledge in this could be due to several reports of the disease in China (7, 8, 24). The study found that only 56.09% of the participants were aware that RT-PCR was the primary diagnostic method, 47.44% were aware that supportive therapy was the primary treatment, and 35.90% were aware that long-lasting immunity could be gained after infection. A study in Italy similarly found that people in non-endemic areas had low awareness of atypical symptoms of mosquito-borne diseases, a common problem that suggests the need to reinforce knowledge about diagnosis and treatment in training (25). Rapid case detection and response is critical to contain local outbreaks (26). Socio-demographic analysis further revealed that physicians had significantly higher knowledge scores than nurses,which was similar to the findings of Menchaca et al. (27). This may be due to the fact that physicians are more educated than nurses overall, and previous studies have shown that education is a key factor in knowledge scores (11, 27–29). Besides, secondary hospital personnel had higher scores than primary hospitals. Clinically, primary healthcare providers often struggle to distinguish CHIKV’s non-specific acute symptoms from those of dengue fever, frequently resulting in misdiagnosis (30). Findings of a study from Ethiopia that specialized training experience enhances knowledge of mosquito-borne infectious diseases (29).

Our study found that respondents had low local risk perception but high willingness to participate in prevention and control. 32.37% of participants perceived a high risk of chikungunya fever locally. Similarly, a study in the Lazio region of Italy found only 15.2% of Italians were concerned about mosquito-caused disease prior to the 2017 chikungunya outbreak (25). However, low risk perceptions may lead to inadequate preparedness for prevention and control, as the risk of local transmission from imported cases in non-endemic areas continues to rise (11). In contrast, the vast majority of respondents showed a positive and proactive attitude: 90.06% were willing to learn about Chikungunya fever on their own, 94.88% recognized the importance of healthcare workers’ knowledge of prevention and control, and 75.64% agreed with the effectiveness of mosquito prevention and control measures. This was consistent with the findings of the Thai study, in which local community residents showed a strong willingness to support new mosquito control methods such as sterile insect techniques, although they had limited knowledge about them (31). Notably, more positive attitudes were observed among those with senior titles and those who had worked for 5–10 years, aligning with findings from Iran (16). This may stem from their accumulated clinical experience with infectious diseases and a habit of searching new facts and updates about emerging and re-emerging epidemics (32).

However, despite better knowledge scores and positive attitudes among healthcare workers, there were deficiencies in practice behaviors. 68.59% of participants would frequently or consistently ask about the travel history of patients with fever with arthralgia, but only 34.97% were able to consistently conduct Chikungunya public health education. Participation in training was also poor, 50.96% of respondents had participated in only 1–2 relevant trainings in the past year, while 11.54% had participated in 3 or more, and 28.85% were willing but unable to obtain training opportunities. Similarly, this results between good knowledge, attitudes and poorer behavior have been observed in other cross-sectional studies or meta-analyses (33–37). Indeed, it will continue to be a principal challenge in managing dengue and Aedes vector populations if the gap is not decreased (38). This problem is not unique and is also evident in the prevention and control of other infectious diseases. For example, studies during the COVID-19 control period have shown that providing healthcare workers with systematic training programs, adequate personal protective equipment, and the opportunity to participate in online seminars is one of the most critical clinical recommendations for improving their knowledge, attitudes, and practices (39–41). These measures are highly consistent with the training needs revealed in this study. Of particular interest is the fact that social networks have been shown to be one of the most important sources of information for improving healthcare workers’ knowledge (42, 43). In the absence of specific drugs, social networks play a central role in disseminating information and facilitating the public’s social distance (44, 45). This provides new ideas for improving the education of neglected infectious diseases such as chikungunya: in the future, in addition to building supportive environments and integrating training into the continuing education system, social media platforms should be actively utilized to develop convenient clinical decision aids and education tools to facilitate healthcare professionals’ translation of knowledge into daily practice in a more efficient and widespread manner.

This study suggest there may be a significant positive correlation between the three KAP components. This finding was the same as a previous cross-sectional study conducted in Ethiopia (29). Similarly, positive associations between knowledge, attitudes, and behavior have been found in other studies of insect-borne infectious diseases (46, 47). The research indicates that data can be translated into a collective mindset and behaviors that foster beneficial routines (29).

The identified KAP gaps and training inadequacies highlight the need for systematic interventions by health authorities (23). In China, it is imperative to increase investment in educational materials to enhance the training coverage and quality of healthcare workers at all levels (11). Second, social media platforms (such as Douyin) should be actively used to facilitate real-time knowledge sharing and case discussions. Additionally, simplified tools should be developed to guide frontline workers in diagnosis, reporting and case management. These coordinated efforts are crucial for building a resilient frontline defense against the growing threat of imported arboviral diseases.

This study has some limitations that should be considered when interpreting the results. First, the cross-sectional design establishes associations between knowledge, attitudes, and practices but does not permit causal inference regarding their directional relationships. Second, reporting bias may exist as our information were collected from a self-designed questionnaire and some respondents may provide responses that conform to social expectations (29, 31). Third, although the questionnaire was developed based on expert consultation and literature review, it was not formally pilot-tested in an external sample. Fourth, the online recruitment method primarily used WeChat, which may have resulted in the underrepresentation of older, less tech-savvy healthcare workers and those in remote or rural settings with limited internet access. Nevertheless, the findings still offer valuable insights for public health policymakers and healthcare administrators, supporting the development of targeted preventive measures against Chikungunya fever. Future studies should employ longitudinal or interventional designs and adopt mixed-mode recruitment strategies to better include under-represented segments of the healthcare workforce, thereby strengthening the evidence base for chikungunya fever preparedness.

## 5 Conclusion

In conclusion, this study demonstrates a critical knowledge-attitude-practice disconnect among healthcare workers in a non-endemic area of China. The identified gaps particularly in diagnostic knowledge, training participation, and public health education delivery provide a crucial evidence base for designing targeted interventions. More importantly, these specific gaps serve as readily measurable metrics for evaluating the effectiveness of future programs. For instance, improvements in correct diagnosis recognition rates and the frequency of public health education can be directly tracked as indicators of intervention success. By focusing resources on the most vulnerable groups (nurses, primary care providers) and addressing the precise deficits revealed in this KAP framework, public health authorities can develop more efficient and evaluable strategies to strengthen frontline preparedness against emerging arboviral threats.

## Data Availability

The raw data supporting the conclusions of this article will be made available by the authors, without undue reservation.
