# Correction: Knowledge, attitudes, and practices regarding chikungunya fever among healthcare workers: a cross-sectional study in Sichuan Province, China

**DOI:** 10.3389/fpubh.2026.1808525

**Published:** 2026-02-20

**Authors:** Frontiers Production Office

**Affiliations:** Frontiers Media SA, Lausanne, Switzerland

**Keywords:** knowledge, attitudes, practices, chikungunya fever, healthcare workers, China

Affiliation “All India Institute of Medical Sciences Bhubaneswar, India” was erroneously given as “All India Institute of Medical Sciences Rishikesh, India” for reviewer Diksha Mohapatra.

The original version of this article has been updated.

