# Supplementary material for: Knowledge, attitudes, and practices regarding chikungunya fever among healthcare workers: a cross-sectional study in Sichuan Province, China
Source: Front Public Health. 2026 Jan 13;13:1729173. doi: 10.3389/fpubh.2025.1729173 (PMC12835402; doi:10.3389/fpubh.2025.1729173)
Supplement: Supplementary file 1 [file Table_1.DOCX]

**Table S1 Knowledge for different characteristics of the population**

| **Variable** | **Poor** | **Good** | **χ²** | **P** |
| --- | --- | --- | --- | --- |
| **Age (Years)** |  |  | 0.714 | 0.870 |
| 19-30 | 60 | 89 |  |  |
| 31-40 | 43 | 74 |  |  |
| 41-50 | 14 | 18 |  |  |
| 51-60 | 5 | 9 |  |  |
| **Gender** |  |  | 0.856 | 0.355 |
| Male | 35 | 64 |  |  |
| Female | 87 | 126 |  |  |
| **Education** |  |  | 0.750 | 0.861 |
| Junior high school | 1 | 2 |  |  |
| Senior/vocational high school | 6 | 10 |  |  |
| Junior college/bachelor's degree | 112 | 170 |  |  |
| Master's degree or above | 3 | 8 |  |  |
| **Occupation** |  |  | 4.458 | 0.035 |
| Nurse | 63 | 75 |  |  |
| Physician | 59 | 115 |  |  |
| **Workplace** |  |  | 8.465 | 0.015 |
| Primary hospital | 75 | 85 |  |  |
| Secondary hospital | 38 | 82 |  |  |
| Tertiary hospital | 9 | 23 |  |  |
| **Professional title** |  |  | 5.274 | 0.153 |
| None | 46 | 62 |  |  |
| Junior | 38 | 48 |  |  |
| Intermediate | 27 | 65 |  |  |
| Senior | 11 | 15 |  |  |
| **Department** |  |  | 4.701 | 0.071 |
| Internal medicine | 37 | 82 |  |  |
| Surgery | 19 | 27 |  |  |
| Public health | 10 | 19 |  |  |
| Others | 56 | 62 |  |  |
| **Working years** |  |  | 5.458 | 0.141 |
| <5 | 42 | 47 |  |  |
| 5-10 | 40 | 82 |  |  |
| 11-15 | 19 | 23 |  |  |
| >15 | 21 | 38 |  |  |

**Table S2 Attitudes for different characteristics of the population**

| **Variable** | **Poor** | **Good** | **χ²** | **P** |
| --- | --- | --- | --- | --- |
| **Age (Years)** |  |  | 6.968 | 0.073 |
| 19-30 | 59 | 90 |  |  |
| 31-40 | 40 | 77 |  |  |
| 41-50 | 5 | 27 |  |  |
| 51-60 | 4 | 10 |  |  |
| **Gender** |  |  | 2.147 | 0.143 |
| Male | 40 | 59 |  |  |
| Female | 68 | 145 |  |  |
| **Education** |  |  | 3.987 | 0.263 |
| Junior high school | 2 | 1 |  |  |
| Senior/vocational high school | 4 | 12 |  |  |
| Junior college/bachelor's degree | 96 | 186 |  |  |
| Master's degree or above | 6 | 5 |  |  |
| **Occupation** |  |  | 0.087 | 0.768 |
| Nurse | 49 | 89 |  |  |
| Physician | 59 | 115 |  |  |
| **Workplace** |  |  | 0.574 | 0.750 |
| Primary hospital | 54 | 106 |  |  |
| Secondary hospital | 41 | 79 |  |  |
| Tertiary hospital | 13 | 19 |  |  |
| **Professional title** |  |  | 10.024 | 0.018 |
| None | 43 | 65 |  |  |
| Junior | 36 | 50 |  |  |
| Intermediate | 20 | 72 |  |  |
| Senior | 9 | 17 |  |  |
| **Department** |  |  | 6.548 | 0.088 |
| Internal medicine | 45 | 74 |  |  |
| Surgery | 15 | 31 |  |  |
| Public health | 4 | 25 |  |  |
| Others | 44 | 74 |  |  |
| **Working years** |  |  | 13.317 | 0.004 |
| <5 | 41 | 48 |  |  |
| 5-10 | 42 | 80 |  |  |
| 11-15 | 15 | 27 |  |  |
| >15 | 10 | 49 |  |  |

**Table S3 Practices for different characteristics of the population**

| **Variable** | **Poor** | **Good** | **χ²** | **P** |
| --- | --- | --- | --- | --- |
| **Age (Years)** |  |  | 0.822 | 0.844 |
| 19-30 | 89 | 60 |  |  |
| 31-40 | 69 | 48 |  |  |
| 41-50 | 19 | 13 |  |  |
| 51-60 | 10 | 4 |  |  |
| **Gender** |  |  | 2.736 | 0.098 |
| Male | 66 | 33 |  |  |
| Female | 121 | 92 |  |  |
| **Education** |  |  | 5.089 | 0.165 |
| Junior high school | 2 | 1 |  |  |
| Senior/vocational high school | 11 | 5 |  |  |
| Junior college/bachelor's degree | 164 | 118 |  |  |
| Master's degree or above | 9 | 2 |  |  |
| **Occupation** |  |  | 0.398 | 0.528 |
| Nurse | 80 | 58 |  |  |
| Physician | 107 | 67 |  |  |
| **Workplace** |  |  | 1.384 | 0.501 |
| Primary hospital | 91 | 69 |  |  |
| Secondary hospital | 75 | 45 |  |  |
| Tertiary hospital | 21 | 11 |  |  |
| **Professional title** |  |  | 1.160 | 0.763 |
| None | 65 | 43 |  |  |
| Junior | 51 | 35 |  |  |
| Intermediate | 53 | 39 |  |  |
| Senior | 18 | 8 |  |  |
| **Department** |  |  | 6.195 | 0.102 |
| Internal medicine | 80 | 39 |  |  |
| Surgery | 28 | 18 |  |  |
| Public health | 13 | 16 |  |  |
| Others | 66 | 52 |  |  |
| **Working years** |  |  | 2.162 | 0.539 |
| <5 | 57 | 32 |  |  |
| 5-10 | 67 | 55 |  |  |
| 11-15 | 26 | 16 |  |  |
| >15 | 37 | 22 |  |  |

**Table S4 Factors associated with knowledge, attitudes scores (Binary logistic regression)**

| **Domain** | **Variable** | **Category** | **OR (95% CI)** | **P** |
| --- | --- | --- | --- | --- |
| Knowledge | Occupation | Physician vs Nurse | 1.607 (1.009 - 2.560) | 0.045 |
|  | Workplace | Secondary vs Primary | 1.901 (1.156 - 3.127) | 0.011 |
|  |  | Tertiary vs Primary | 1.450 (0.680 - 3.090) | 0.335 |
| Attitudes | Professional title | Senior vs None | 2.099 (1.016 - 4.337) | 0.045 |
|  |  | Intermediate vs None | 1.450 (0.860 - 2.445) | 0.163 |
|  |  | Junior vs None | 1.280 (0.730 - 2.245) | 0.386 |
|  | Working years | 5-10 vs <5 | 4.438 (1.627 - 12.100) | 0.004 |
|  |  | 11-15 vs <5 | 2.010 (0.650 - 6.220) | 0.226 |
|  |  | >15 vs <5 | 2.150 (0.790 - 5.840) | 0.134 |
